# Sequencing and analysis of the complete chloroplast genome of the traditional medicinal plant *Fraxinus chinensis* subsp. *rhynchophylla* (Oleaceae)

**DOI:** 10.1080/23802359.2025.2594311

**Published:** 2025-12-03

**Authors:** Yan-liang Guo, Yu-tong Huang, Yan-ping Xing, Ting-guo Kang, Yan-yun Yang, Liang Xu

**Affiliations:** ^a^ School of Pharmacy, Liaoning University of Traditional Chinese Medicine, Dalian, China; ^b^ Traditional Chinese Medicine Quality and Resource Development Professional Technology Innovation Center, Liaoning Provincial Department of Science and Technology, Shenyang, China

**Keywords:** *Fraxinus chinensis* subsp. *rhynchophylla*, chloroplast genome, phylogenetic tree, analysis

## Abstract

*Fraxinus chinensis* subsp.* rhynchophylla* is a traditional medicinal plant. Its chloroplast genome was sequenced, assembled, and analyzed in this study. The results showed that the complete chloroplast genome of* F. chinensis* subsp.* Rhynchophylla,* 155,784 bp in length, contains 133 annotated genes, including 88 protein-coding genes (PCGs), 8 rRNA genes, and 37 tRNA genes. Phylogenetic analysis revealed that this subspecies clusters with 14 other *Fraxinus* species, such as* Fraxinus sieboldiana* and* Fraxinus hupehensis*, with strong support values. These findings provide a valuable resource for further evolutionary studies, molecular identification, and medicinal applications of* F. chinensis* subsp.* rhynchophylla.*

## Introduction

*Fraxinus chinensis* subsp. *rhynchophylla* (Hance) E. Murray (1869) is a deciduous tree belonging to the genus *Fraxinus* within the Oleaceae family. It is primarily distributed in Northern China, Russia, and Korea. The dried stem bark and branch bark of *F. chinensis* subsp. *rhynchophylla* (*Fraxinus chinensis* Hance) serve as one of the botanical sources for the traditional Chinese medicine Qinpi (*Cortex Fraxini*) (Chang et al. 1996; Wallander 2008; Chinese Pharmacopoeia Commission 2020). Modern pharmacological studies have demonstrated that it possesses anti-inflammatory, antibacterial, and antioxidant properties (Fang et al. 2008). Additionally, this plant is also valued as an ornamental tree with considerable economic importance (An et al. 2022). However, the lack of chloroplast genome information has constrained its further development and utilization.

Chloroplast genome, which typically exhibits a quadripartite structure (Zhu et al. 2016), is now extensively employed in phylogenetic research owing to its structural simplicity and high stability (Li et al. 2025). Now, the chloroplast genomes of closely related species of *Fraxinus chinensis* subsp. *rhynchophylla* have been successively reported, such as *Fraxinus chinensis* and *Fraxinus angustifolia*. They exhibit highly similar structural features and sizes, including the typical quadripartite structure, a GC content of approximately 37%, and a total gene number of around 130 (Olofsson et al. 2019). Moreover, in numerous medicinal plants, species-specific DNA barcodes derived from chloroplast genome sequences have been developed to facilitate accurate botanical identification and source authentication (Li et al. 2015). Prominent examples include *Bupleurum chinense* and *Panax ginseng* (Zuo et al. 2011; Chao et al. 2014).

Therefore, we sequenced and analyzed the complete chloroplast genome of *F. chinensis* subsp. *rhynchophylla* to characterize its structural features, gene composition, and phylogenetic relationships. The resulting genomic data provide valuable insights for comparative and phylogenetic analyses within *Fraxinus* and offer a molecular basis for the identification and utilization of this species.

## Materials and methods

### Plant material

**Figure 1.**
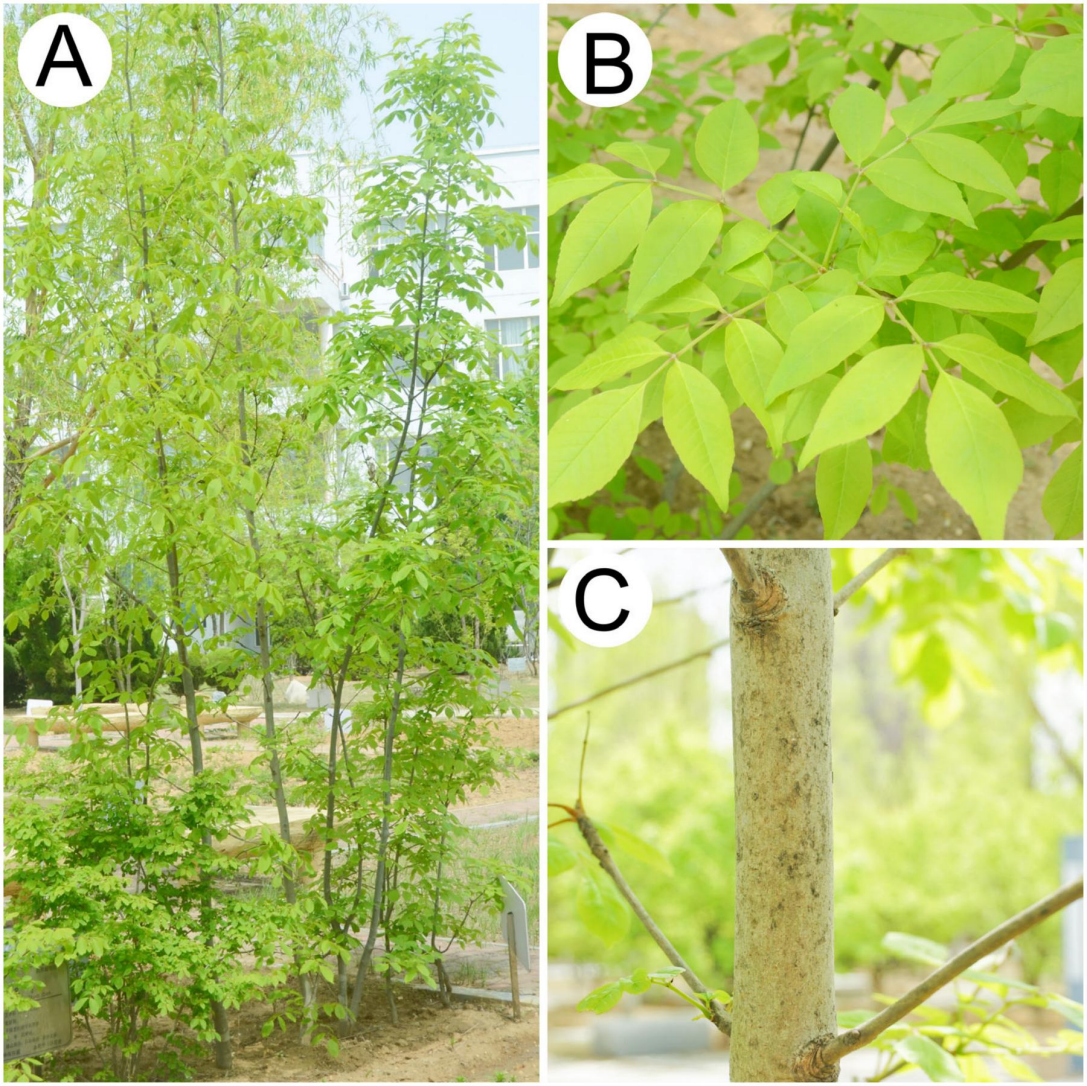
Photographs of (A) habit, (B) leaves, and (C) stem of *F. chinensis* subsp. *rhynchophylla* taken by Huang Yu-tong in Dalian, Liaoning Province, China (121°52′56.04″ E, 39°03′49.89″ N). It is a deciduous tree. Leaves: pinnately compound, 15–35 cm long. Petiole: base swollen. Leaflets: 3–7, terminal leaflet broadly ovate to elliptic, sometimes lanceolate. Bark: grayish-brown, smooth.

Fresh leaf samples were collected from Dalian, Liaoning Province, China (121°52′56.04″ E, 39°03′49.89″ N) and identified by Professor Liang Xu from the Liaoning University of Traditional Chinese Medicine (Figure 1). The specimen was deposited at the herbarium of Liaoning University of Traditional Chinese Medicine (Liang Xu 861364054@qq.com, voucher code: 10162240512002LY) (Supplementary Figure S1).

### DNA extraction and sequencing

Total genomic DNA was extracted from 150 mg of fresh leaves using cetyltrimethylammonium bromide (CTAB) method (Doyle 1987). DNA quality was verified by 1% agarose gel electrophoresis, and concentration was measured with a Qubit^®^ 3.0 fluorometer (Invitrogen, Carlsbad, CA). Purified DNA (1 μg) was sheared to ∼350 bp by ultrasonication, and a sequencing library was prepared from these fragments using the Illumina Nextera XT kit (Illumina, San Diego, CA). Paired-end sequencing was performed on the Illumina NovaSeq 6000 platform, and sequencing depth was calculated with samtools depth (Li et al. 2009).

### Genome assembly and annotation

Raw reads (4.81 Gb) were quality-filtered with the NGS QC Toolkit v2.3.3 (Patel and Jain 2012), yielding 4.74 Gb of clean data. *De novo* assembly was conducted with SPAdes v3.14.1 (Bankevich et al. 2012). Genome annotation was performed using Plastid Genome Annotator (PGA) (Qu et al. 2019), with *Fraxinus sieboldiana* (MK299395) as the reference, followed by manual inspection and correction. Chloroplast genome maps and visualization of cis- and trans-spliced genes were generated using CPGview (http://www.1kmpg.cn/cpgview) (Liu et al. 2023).

### Phylogenetic analysis

To determine the phylogenetic position of *F. chinensis* subsp. *rhynchophylla* within the Oleaceae, 34 species from 12 genera with complete chloroplast genomes available in NCBI were selected. *Paulownia fortunei* (NC045087) served as the outgroup. Sixty-one shared protein-coding genes (PCGs) from 36 taxa were aligned using MAFFT v7.429 and refined with Gblocks 0.91b to remove poorly aligned regions (Kück et al. 2010; Katoh and Standley 2013). The concatenated alignment was used to construct a maximum-likelihood (ML) tree under the TVM + F + R2 model with 1000 bootstrap replicates.

## Results

### Genome structure analysis

**Figure 2.**
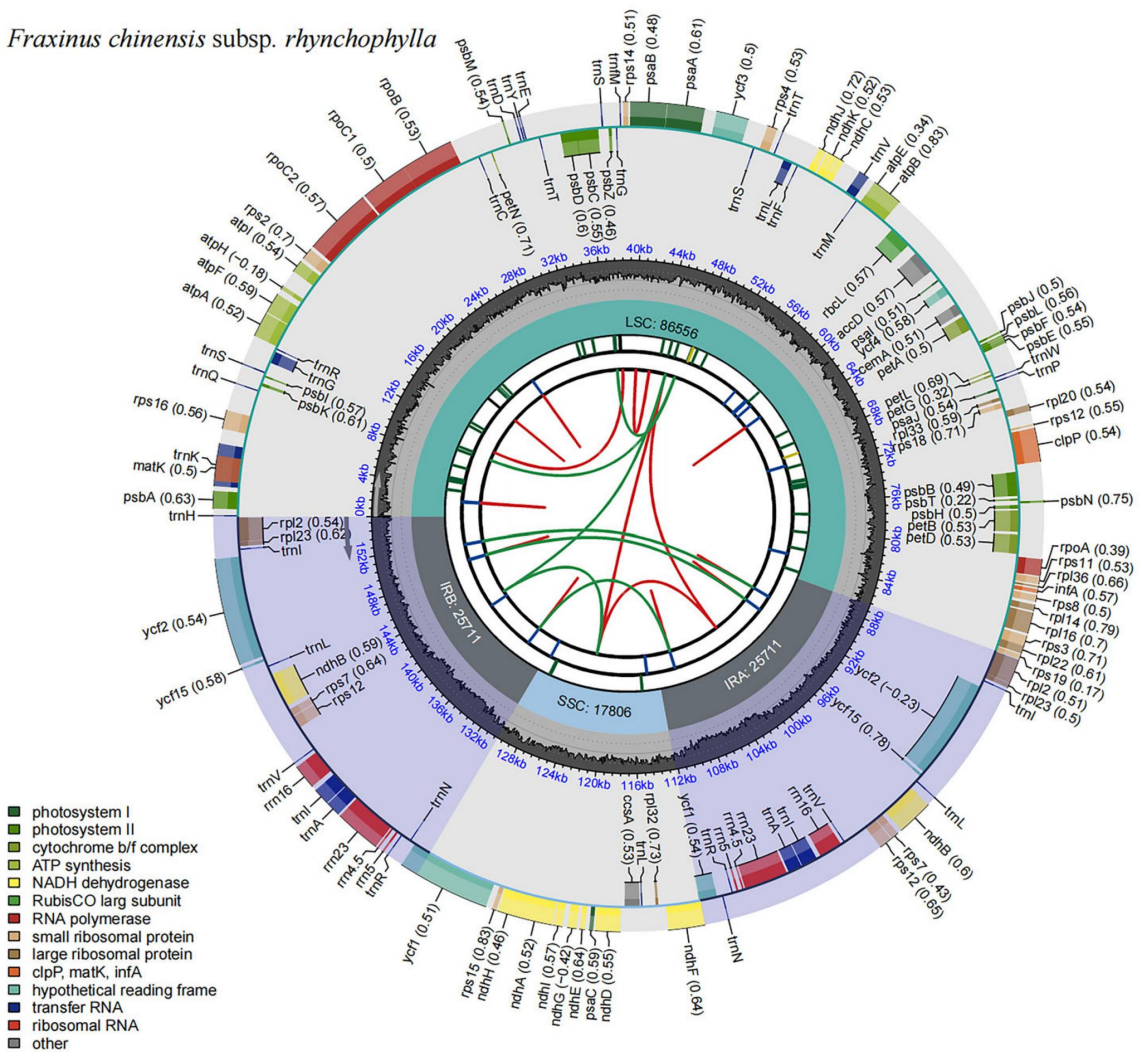
The schematic representation of the *F. chinensis* subsp. *rhynchophylla* chloroplast genome. Genes drawn outside the circle are transcribed clockwise, and those inside are counterclockwise. The map consists of several circles, each with the following information from the center outward: innermost circles: red and green arcs representing direct (D) and palindromic (P) repeats, respectively. Next two circles: short bars indicating tandem repeats and microsatellite sequences, respectively. Fourth circle: positions of the LSC, SSC, IRA, and IRB regions. Fifth circle: GC (darker gray) and AT (lighter gray) content. Outer circle: gene functions, with different colors denoting functional categories, as shown in the bottom left corner.

The assembled chloroplast genome has a total length of 155,784 bp and a GC content of 37.86%, displaying the characteristic quadripartite structure (Figure 2). The average sequencing depth was 1365.79 ×, ranging from the minimum of 470× to the maximum of 2115× (Supplementary Figure S2).

Annotation identified a total of 133 genes, including 88 PCGs, eight rRNA genes, and 37 tRNA genes. Fifteen genes (*atp*F, *ndh*A, *ndh*B, *pet*B, *pet*D, *rpl*16, *rpl*2, *rpo*C1, *rps*16, *trn*A-UGC, *trn*G-UCC, *trn*I-GAU, *trn*K-UUU, *trn*L-UAA, *trn*V-UAC) each contain one intron. Two genes (*clp*P and *ycf*3) each possess two introns. The *rps*12 gene exhibits trans-splicing (Supplementary Figures S3 and S4).

### Phylogenetic analysis

**Figure 3.**
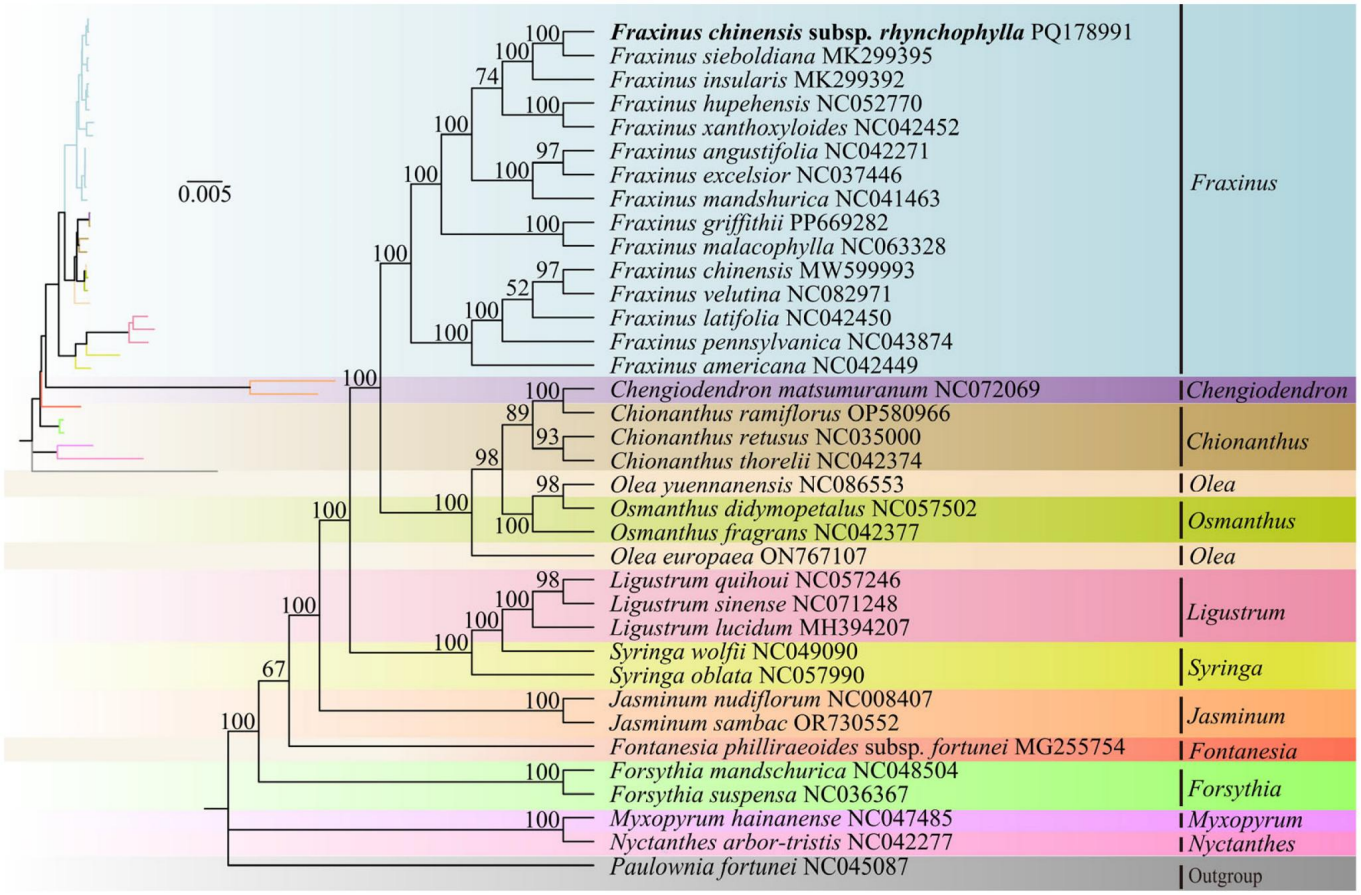
ML phylogenetic tree of *F. chinensis* subsp. *rhynchophylla* based on 61 shared PCGs from this taxon and 35 additional species. Numbers above branches indicate ML bootstrap support values. TVM + F + R2 was identified as the optimal substitution model by ModelFinder. The scale bar in the top-left corner of the figure represents the evolutionary distance, with a unit length of 0.005. Reference sequences include: *Fraxinus chinensis* MW599993, *Fraxinus griffithii* PP669282, *Fraxinus malacophylla* NC063328 (Duan et al. 2020), *Fraxinus pennsylvanica* NC043874, *Fraxinus hupehensis* NC052770 (Zhang et al. 2020), *Fraxinus excelsior* NC037446, *Fraxinus velutina* NC082971, *Fraxinus americana* NC042449 (Olofsson et al. 2019), *Fraxinus angustifolia* NC042271 (Olofsson et al. 2019), *Fraxinus insularis* MK299392 (Olofsson et al. 2019), *Fraxinus latifolia* NC042450 (Olofsson et al. 2019), *Fraxinus mandshurica* NC041463, *Fraxinus. sieboldiana* MK299395 (Olofsson et al. 2019), *Fraxinus xanthoxyloides* NC042452 (Olofsson et al. 2019), *Olea europaea* ON767107, *Olea yuennanensis* NC086553, *Ligustrum lucidum* MH394207 (Wang et al. 2018), *Ligustrum sinense* NC071248 (Long et al. 2023), *Ligustrum quihoui* NC057246 (Wang et al. 2019), *Osmanthus fragrans* NC042377, *Osmanthus matsumuranus* NC072069, *Osmanthus didymopetalus* NC057502 (Zhao et al. 2020), *Chionanthus guangxiensis* NC042452 (Olofsson et al. 2019), *Chionanthus ramiflorus* OP580966, *Forsythia suspensa* NC036367 (Wang et al. 2017), *Forsythia mandschurica* NC048504 (Olofsson et al. 2019), *Syringa wolfii* NC049090 (Liu et al. 2020), *Syringa oblata* NC057990, *Jasminum sambac* OR730552, *Jasminum nudiflorum* NC008407 (Lee et al. 2007), *Chionanthus retusus* NC035000 (He et al. 2017), *Myxopyrum hainanense* NC042277 (Olofsson et al. 2019), *Fontanesia philliraeoides* subsp. *fortune* MG255754, *Nyctanthes arbor*-*tristis* NC042277 (Olofsson et al. 2019), and outgroup: *Paulownia fortune*i NC045087.

The phylogenetic tree illustrates the evolutionary relationships between *F. chinensis* subsp. *rhynchophylla* and 34 representative Oleaceae species. Most branches showed high bootstrap support. *F. chinensis* subsp. *rhynchophylla* clustered in a well-supported clade with 14 other Fraxinus species, including *F. malacophylla* and *F. hupehensis*, indicating close phylogenetic affinity. Notably, *F. chinensis* subsp. *rhynchophylla* and *F. sieboldiana* were resolved as sister species, indicating their closest phylogenetic relationship within the genus (Figure 3).

## Conclusions and discussion

Compared with previous studies, this work first reports the complete chloroplast genome of *F. chinensis* subsp. *rhynchophylla* and compares it with *F. chinensis*, *F. hupehensis*, and *F. sieboldiana*. The genome exhibits the typical quadripartite structure of most plants (Zhu et al. 2016) and shows high similarity to its relatives in length, GC content, and gene number, with no distinct structural variations, indicating strong conservation within the genus *Fraxinus* (Supplementary Table 1) (Wang et al. 2024, 2025).

Furthermore, this study employed shared chloroplast PCGs to investigate the phylogenetic position of *F. chinensis* subsp. *rhynchophylla* within the Oleaceae family. The phylogenetic analysis of the *Fraxinus* genus included five sections, encompassing the Sect. Ornus and Sect. Ornaster groups. Overall, *F. chinensis* subsp. *rhynchophylla* clustered into a major branch with other *Fraxinus* species, and this clustering pattern is consistent with the independent phylogenetic study based on chloroplast PCGs shared by *Fraxinus* species (Olofsson et al. 2019). Within this cluster, *F. chinensis* subsp. *rhynchophylla* is most closely related to *F. sieboldiana*, which further supports their modern taxonomic classification within the Sect. Ornus, distinguishing them from the traditional morphological classification where *F. chinensis* subsp. *rhynchophylla* is placed in Sect. Ornaster (Chang et al. 1996). Additionally, similar results have been observed in some studies using ITS sequences and phylogenetic trees constructed with three chloroplast genes (*psb*A-*trn*H, *rpl*32-*trn*L, and *mat*K) (Wallander 2008; Wei et al. 2024).

Qinpi is frequently adulterated with products such as the dried bark of *Juglans mandshurica* and *Albizia julibrissin*. Accurate molecular identification is therefore essential. Previous classification methods relied mainly on morphological and spectroscopic features (Wu 1983; Wu et al. 1983). Among the four official Qinpi source species, only *F. chinensis* and now *F. chinensis* subsp. *rhynchophylla* have publicly available chloroplast genomes, providing crucial data for developing genomic identification methods.

In summary, this study enhances understanding of *Fraxinus* phylogeny and offers essential genomic resources for species identification, authentication of Qinpi, and future research on the medicinal potential of *F. chinensis* subsp. *rhynchophylla*.

## Supplementary Material

A Clean Supplementary materials.docx

Supplementary materials with track.docx

## Data Availability

The genome sequence data that support the findings of this study are openly available in GenBank of NCBI at (https://www.ncbi.nlm.nih.gov/) under accession no. PQ178991. The associated BioProject, SRA, and Bio-Sample numbers are PRJNA1141200, SRR30015775 (Illumina), and SAMN42883145, respectively.
