# Supplementary material for: Sequencing and analysis of the complete chloroplast genome of the traditional medicinal plant Fraxinus chinensis subsp. rhynchophylla (Oleaceae)
Source: Mitochondrial DNA B Resour. 2025 Dec 3;11(1):17–22. doi: 10.1080/23802359.2025.2594311 (PMC12677035; doi:10.1080/23802359.2025.2594311)
Supplement: A Clean Supplementary materials.docx [file TMDN_A_2594311_SM1392.docx]

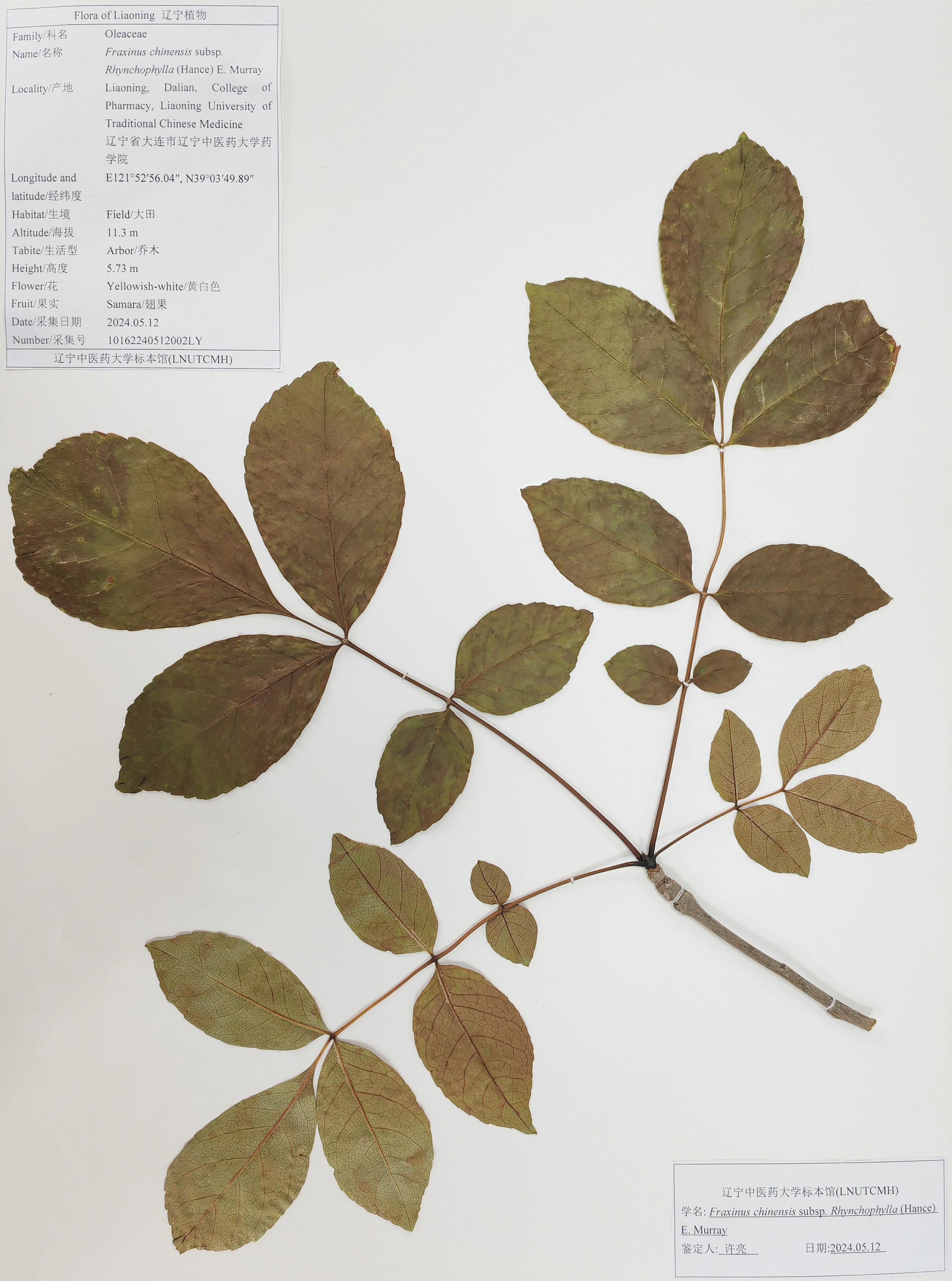


**Figure S1.** An illustration of *Fraxinus chinensis* subsp. *rhynchophylla* (Hance) E. Murray specimen preserved at Liaoning University of Traditional Chinese Medicine. Specimen number, collector, latitude and longitude and other details are noted on the map for reference.


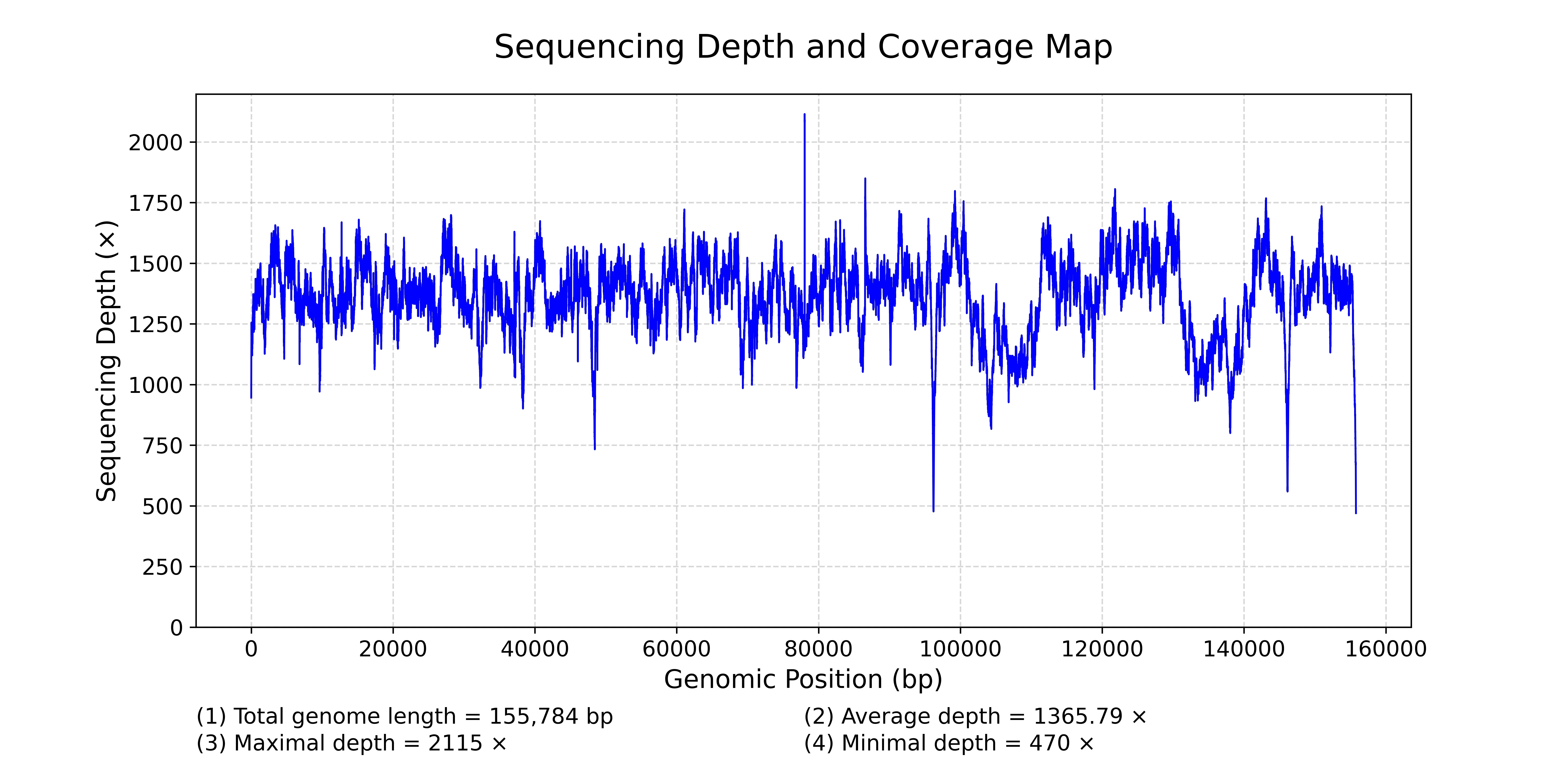


**Figure S2.** Sequencing coverage depth of *Fraxinus chinensis* subsp. *rhynchophylla* (Hance) E. Murray.The illumina short sequences were compared to the chloroplast genome sequences using BWA software and finally the coverage was calculated using samtools depth (The maximum sequencing depth was 2115 ×, the minimum sequencing depth was 470 ×, and the average sequencing depth was 1365.79 ×). The horizontal coordinate is the chloroplast length and the vertical coordinate is the coverage depth.

Li, H. 2013. Aligning sequence reads, clone sequences and assembly contigs with BWA-MEM. arXiv: Genomics.

Li, H., Handsaker, B., Wysoker, A., Fennell, T., Ruan, J., Homer, N., Marth, G., Abecasis, G., Durbin, R., & 1000 Genome Project Data Processing Subgroup. 2009. The Sequence Alignment/Map format and SAMtools. Bioinformatics (Oxford, England), 25(16), 2078–2079.


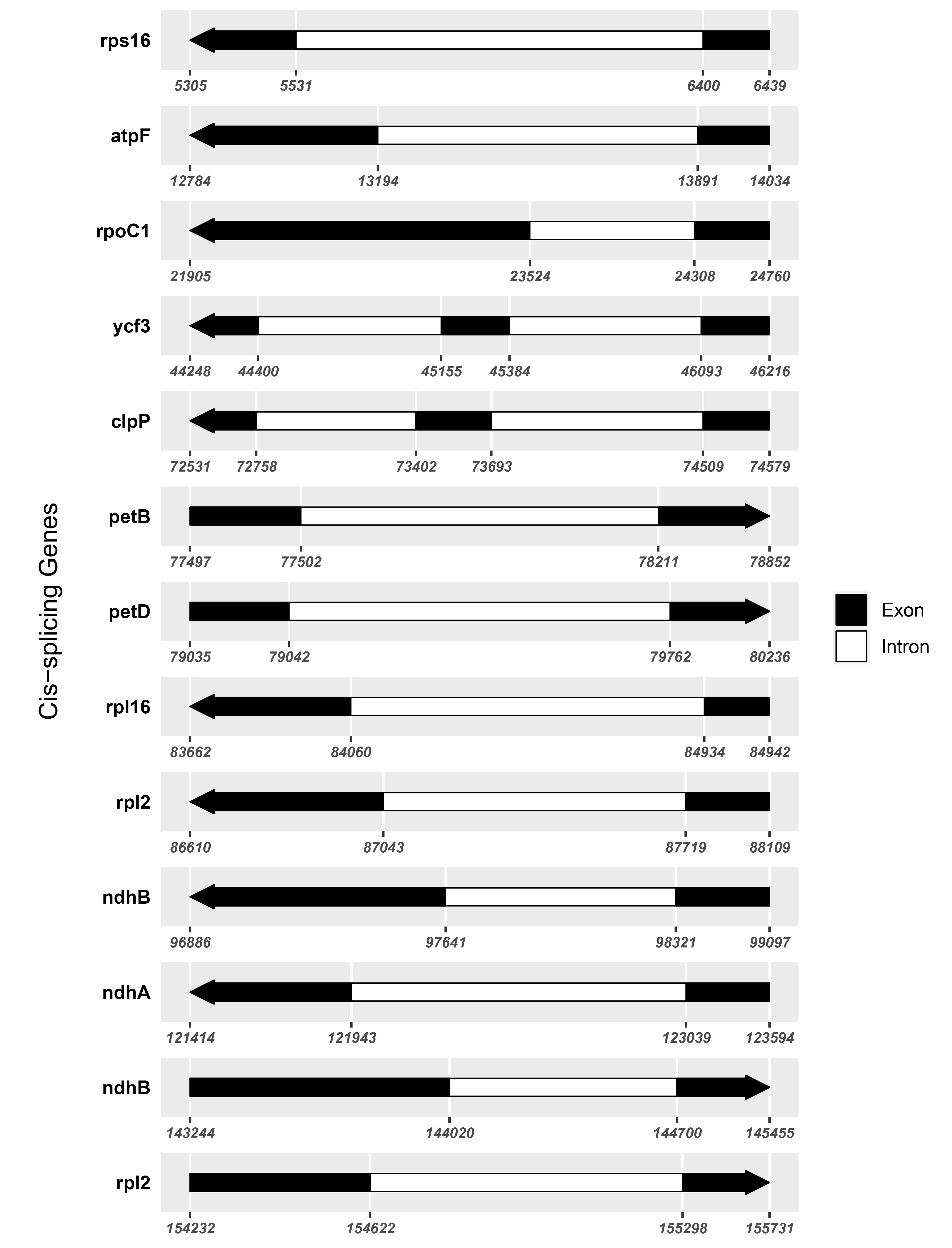


**Figure S3.** Schematic map of the cis-splicing genes in the chloroplast genome. The genes are arranged from top to bottom based on their order on the chloroplast genome. The gene names are shown on the left, and the gene structures are on the right. The exons are shown in black; the introns are shown in white. The arrow indicates the sense direction of the gene. Please note that lengths of exons and introns are not drawn to scale.


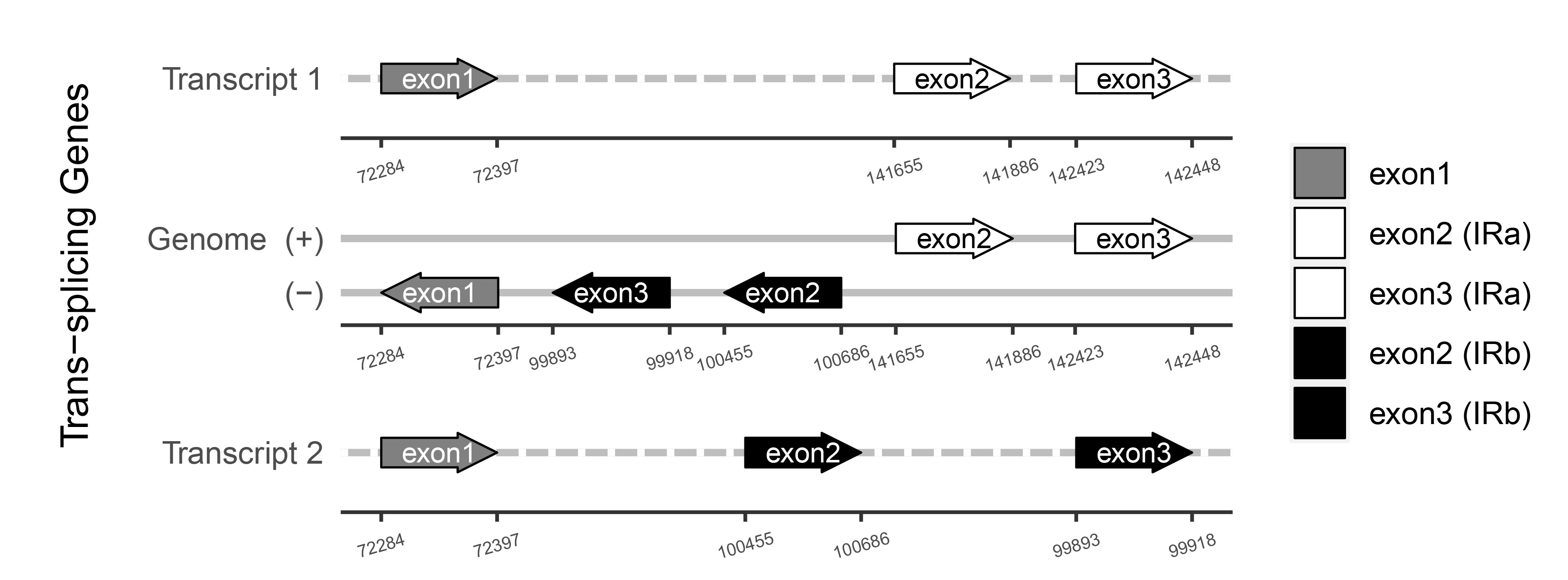


**Figure S4.** Schematic map of the trans-splicing gene *rps*12 in the chloroplast genome. It has three unique exons. Two of them are duplicated as they are located in the IR regions.

**Table S1** Comparison Table of Chloroplast Genome Characteristics of *Fraxinus chinensis* subsp. *rhynchophylla*, *Fraxinus chinensis*, *Fraxinus hupehensis* and *Fraxinus sieboldiana*.

| **Genome feature** | ***Fraxinus chinensis* subsp. *rhynchophylla*** | ***Fraxinus chinensis*** | ***Fraxinus hupehensis*** | ***Fraxinus sieboldiana*** |
| --- | --- | --- | --- | --- |
| Genome size (bp) | 155784 | 155610 | 155689 | 155703 |
| LSC length (bp) | 86556 | 86486 | 86498 | 86475 |
| SSC length (bp) | 17806 | 17760 | 17967 | 17804 |
| IR length (bp) | 25711 | 25682 | 25612 | 25712 |
| GC content (%) |  |  |  |  |
| Total genome | 37.86 | 37.84 | 37.85 | 37.86 |
| LSC | 35.87 | 35.84 | 35.87 | 35.88 |
| SSC | 31.99 | 32.02 | 31.91 | 32.03 |
| IR | 43.23 | 43.22 | 43.27 | 43.22 |
| Genes (total/different) | 133/115 | 131/113 | 131/113 | 131/113 |
| CDS (total/different) | 88/81 | 88/81 | 88/81 | 88/81 |
| tRNA (total/different) | 37/30 | 35/28 | 35/28 | 35/28 |
| rRNA (total/different) | 8/4 | 8/4 | 8/4 | 8/4 |
| Genes with introns | 23 | 21 | 21 | 21 |
| Different CDS in LSC | 61 | 61 | 61 | 61 |
| Different CDS in SSC | 11 | 11 | 11 | 11 |
| Different CDS in IRB | 7 | 7 | 7 | 7 |
| Different CDS in IRA | 7 | 7 | 7 | 7 |
| Different CDS in LSC/IRB | 0 | 0 | 0 | 0 |
| Different CDS in IRB/SSC | 1 | 1 | 1 | 1 |
| Different CDS in SSC/IRA | 1 | 1 | 1 | 1 |
| Different CDS in IRA/LSC | 0 | 0 | 0 | 0 |
| GeneBank accession | PQ178991 | MW599993 | NC052770 | MK299395 |

Note: Different CDS: The number of non-redundant protein coding genes.
